# Optimizing cholecystectomy time in moderate acute biliary pancreatitis: A randomized clinical trial study

**DOI:** 10.1016/j.heliyon.2020.e03388

**Published:** 2020-02-17

**Authors:** Abdoulhossein Davoodabadi, Esmail Beigmohammadi, Hamidreza Gilasi, Abbas Arj, Hossein Taheri nassaj

**Affiliations:** a Departments of surgery, Trauma Research Center, Kashan University of Medical Sciences, Kashan, Iran; b Departments of Epidemiology& Biostatistics, Kashan University of Medical Sciences, Kashan, Iran; c Department of Internal Medicine, Kashan University of Medical Sciences, Kashan, Iran; d Departments of surgery, Kashan University of Medical Sciences, Kashan, Iran

**Keywords:** Biological sciences, Health sciences, Surgery, Internal medicine, Acute biliary pancreatitis, Laparoscopic cholecystectomy, Mild to moderate acute biliary pancreatitis

## Abstract

**Background:**

In mild to moderate gallstone pancreatitis, cholecystectomy is the most appropriate treatment for prevention of further biliary attacks. However, the timing of cholecystectomy is not precisely determined. The present study was conducted to compare outcomes of very early (within 48 h) versus delayed (more than 1 week) laparoscopic cholecystectomy in patients with acute biliary pancreatitis (ABP).

**Methods:**

This randomized clinical trial study was conducted in Shahid Beheshti Hospital of Kashan University of Medical Sciences from September 2016 to Mar 2019. Two hundred and eight cases with mild to moderate ABP were randomly assigned to 2 groups, with 104 patients in group 1 (operation within 48 h) and 104 in group 2 (operation after one week). Age, sex, biochemical parameters, clinical manifestation at the time of admission, operation time, recurrent biliary problems, relapse, peri-operative complications, conversion rate, and hospital length of stay in the two groups were recorded and compared. In addition, Ranson's score and Revised Atlanta criteria, the American Society of Anaesthesiologists Physical Status ASA-PS, Charlson Co-Morbidity Index (CCI), complexity of surgery and Clavien-Dindo score were also determined.

**Results:**

There were no differences in demographics, peri-operative complications 4 (4%) vs. 4 (4%), P = 1), conversion rate (10.6% vs. 11.5%; P = 0.825) and procedure time (83 vs. 81 minutes, P = 0.110) between the two groups. There were no deaths in either group; however, the length of hospital stay was shorter in the early group compared to the delayed one, (3.66 ± 1.12 vs. 10.35 ± 1.76, P < 0.001).

**Conclusion:**

Cholecystectomy within 48 h decreases significantly the length of hospital stay, without any difference in conversion rate, procedure time, or complication rate.

## 1 Introduction

Acute biliary pancreatitis (ABP) is a disease, most commonly due to biliary calculi, followed by alcohol intake (19.7%) [1]. In 80% of cases it has a mild to moderate course but in the remaining 20% it is accompanied by severe pancreatitis, which may be associated with high morbidity and mortality [2].

Although there is a general agreement about delayed cholecystectomy in severe pancreatitis, until the inflammatory process subsided [3], but in mild to moderate gallstone pancreatitis, in which cholecystectomy is mandatory to prevent further biliary events, acute cholecystitis, cholangitis and biliary colic [4], the precise timing of the procedure is still controversial [5].

In gallstone pancreatitis, surgeons prefer to postpone surgery until assuring about the safety of an early cholecystectomy, so, they perform it after relief of abdominal pain, recovery of the inflammatory process and normalization of liver function tests.

Van Baal MC [6]et al, recommended performing cholecystectomy during the same hospital admission, Uhl Wet al, recommended it immediately after recovery of pancreatitis attack [7].

The British Society of Gastroenterology recommends cholecystectomy during the same hospital admission but no more than 2 weeks after discharge [8], whereas the American College of Gastroenterology recommends performing cholecystectomy during index admission [9].

Shir Li Jee also recommended a median interval time of 6 days from the diagnosis of ABP as the best time for cholecystectomy [10]. Different recommendations about the cholecystectomy timing between investigations and guidelines arise from differing viewpoints and practical experiments. It may also be due to inadequate prospective randomized controlled clinical trials, addressing the precise timing and safety of cholecystectomy. There is also a general consensus about not to delay cholecystectomy more than 2weeks, because of the possibility of recurrent biliary events, which may be life threatening.

Considering 31% recurrences within 2 weeks after discharge, Ito et al, supposed that a 2 weeks interval may be too long [11]. In a study by Aboulian et al, [12], intervention during 48 h of admission, without improvement in abdominal pain or laboratory abnormalities, resulted in a shorter length of hospital stay and no technical difficulty of the procedure or peri-operative complication rate. Although this approach should be further evaluated by high quality studies, it seems that increment in hospital stay is not necessary.

In addition, standard of care has been improved and now trends are toward performing earlier cholecystectomy.

With the consideration that even an interval of 3–6 days as early cholecystectomy, which was recommended in previous studies, is still too late; this study was conducted to compare the results of very early (within 48 h), versus delayed cholecystectomy (more than 1 week), in mild to moderate acute biliary pancreatitis.

## 2 Methods

This randomized clinical trial study was carried out in Shahid Beheshti Hospital of KAUMS, during September 2016 to Mar 2019. Patients with the diagnosis of primary episode of mild to moderate ABP were allocated randomly into early (within 48 h) and delayed groups (more than 1 week) for cholecystectomy. Demographic data, duration of procedure, events, relapse, peri-operative complications, conversion rate, recurrent biliary and total hospital length of stay were recorded and compared between two groups.

### 2.1 Definition of moderate gallstone pancreatitis

On the base of Ranson's criteria [13], serum aspartate aminotransferase (AST) or, serum Lactate (LDH) of more than three times of the upper limit of normal value, age of less than 70 years, WBC less than 18,000/μL, and blood glucose less than 220 mg/dL on admission were considered as the inclusion criteria. On the base of Revised Atlanta criteria [14]none of the patients had organ failure or systemic complications, although a few in both groups had transient renal insufficiency which improved soon after rehydration without any sequel or mortality, and discharged within the 1st week after operation. Therefore, we considered them as moderate acute biliary pancreatitis, although, still is a debate to be considered as category of moderately severe acute pancreatitis.

### 2.2 Diagnosis of acute pancreatitis

On base of Revised Atlanta criteria diagnosis of acute pancreatitis was made in the presence of abdominal pain suggestive of pancreatitis, serum amylase more than three times of the upper normal value or lipase level more than three times the upper normal value [14].If the diagnosis was in doubt, CT-scan was used to confirm acute pancreatitis. Additionally, the diagnosis of gall stone pancreatitis was also considered if sandy stone in gallbladder was seen on abdominal ultrasonography. In the absence of a CBD stone, but suspected cholestasis (bilirubin≤4 mg/dl without cholangitis and dilatation of the biliary tree, repeated ultrasonography (US) or magnetic resonance cholangiopancreatography (MRCP), was also performed. C reactive protein (CRP) level was used in both groups to monitor disease progress. If CRP level was not decreasing or when it reached >150 mg/dL, a contrast-enhanced abdominal computerized tomography (CT) scan was performed to assess any possible necrotizing pancreatitis.

The majority of our patients meet the criteria for acute pancreatitis on the basis of clinical presentation, laboratory results and (US). CT scan was used only when CRP level was not decreasing or when it reached >150.Our current approach in ABP was using MRCP and US instead of Intra-operative cholangiography. Patients who had no CBD stone, only underwent cholecystectomy but in patients with documented CBD stone, endoscopic retrograde cholangiopancreatography (ERCP) and endoscopic sphincterotomy (ES) were performed immediately after discharge. Preoperative ERCP was limited to patients with cholestasis (bilirubin≤4and CBD dilation. Cholecystectomy for mild to moderate gallstone pancreatitis in group 1 was performed before normalization of serum pancreatic enzymes or resolution of abdominal symptom, but in group2 it was carried out after them.

### 2.3 Study design and randomization

Patients were randomly allocated into either a very early group (within 2days) or group1 (G1) and late (more than 1week) or group2 (G2). Informed consent was obtained from all of the participants.

Some of the patients required additional work up such as cardiologic assessment, pre-cholecystectomy ERCP and discontinuation of clopidogrel, so they operated in the same admission time and considered as group 2. This group included 27% of patients but the majority of them had the criteria of group 1, without additional risk factor. In group1, no additional work up was needed, and so immediately underwent cholecystectomy after obtaining informed consent, regardless of the resolution of abdominal pain or pancreatic enzyme.

We applied The American Society of Anaesthesiologists Physical Status ASA-PS classification [14, 15] and Charlson Comorbidity Index (CCI) to assess patients' physical condition and prognostic criteria of outcome before surgery [16, 17]. We also used Complexity of surgery classification according to the Austrian Chamber of Physician [18], and Clavien-Dindo classification of surgical complications to assess postoperative complications after surgery [19].

### 2.4 Sample size calculation

For showing a reduction of recurrent biliary events and complication with power of 90%- and a two-sided test of 5%, 104 patients had to be included in each group (a total of 208 patients).

### 2.5 Inclusion criteria

In this study, adult non-pregnant patients (age range, 18–69 years) with mild to moderate ABP based on Ranson's score≤ 3 and Revised Atlanta Criteria were included.

Exclusion criteria: Patients diagnosed with severe pancreatitis (Ranson's score ≥3 or persistent organ failure), multiple organ failure [14], necrotizing pancreatitis, and concomitant acute cholangitis, at the time of admission, were excluded.

## 3 Ethical consideration

This study was approved by Kashan University of Medical Sciences (KAUMS) ethics committee, http://ethics.research.ac.ir/IR.KAUMS.MEDNT.REC.1397.006, WHO IRCT (Iranian Registry of Clinical trial number), IRCT20180205038627N2, and was reported in line with the CONSORT 2010 checklist of information. For all participants in each group, the possible complications, advantages and disadvantages of early or delayed intervention were explained and a written informed consent was obtained.

## 4 Data collection

After approval of the study and obtaining a written informed consent from each patient, demographic characteristics, laboratory values and peri-operative events were recorded.

Statistical analysis: SPSS version 16 was used for data analysis. Results were presented as mean ± SD with 95%confidence interval (CI). Independent t-tests were used to assess significant differences between the two groups. Frequencies were presented for categorical variables, and chi-square and Fisher's exact tests for qualitative data. Statistical significance was set at a P value of <0.05.

### 4.1 Operation

All patients received appropriate pri-operative antibiotic prophylaxis. Cholecystectomy was performed in standard manner. Majority of the patients were transferred to ward after surgery and discharged during 48 h, however a few needed to stay in a high-dependency unit bed (HDU) for better medical care and seldom required ICU care in first 24 h.

### 4.2 Follow-up

After hospital discharge, patients were visited in the outpatient clinic 1, 2 and 4 month after discharge.

## 5 Results

The age range was 18–69years with a median of 37.00 ± 11.30 in G1 and 35.48 ± 10.83 in G2 (Table 1). There was no significant difference between the two groups regarding age, gender and duration of procedure. Duration of hospital stay was significantly longer in G2 (10.35 ± 1.76 vs. 3.66 ± 1.12, P < 0.001), (Table 1).

**Table 1:** Demographic data related to timing of cholecystectomy.

variable	Very early group (G1)	Late group *(G2)*	P value∗
	n = 104	n=(104 (	
age	37.00 ± 11.30	35.48 ± 10.83	0.324
Sex			
Male	43 (41.3%)	49 (47.1%)	0.402
Female	61 (58.7%)	55 (52.9%)	
Duration of procedure (minutes)	83.7 ± .7.49	81.73 ± 10.43	0.110
Hospital stay days	3.66 ± 1.12	10.35 ± 1.76	<0. 001
Recurrent biliary events	0 (0%)	4 (4%)	0.121∗

** Chi-square test.

∗ T test.

Majority of our patients (73%) were normal healthy people (ASA I) and the rest were in ASA II class, such as controlled diabetes mellitus (DM) or chronic obstructive pulmonary disease (COPD). No patients had active hepatitis. In the pre-operative cardiology consultation, low to moderate risk and ejection fractions of 45–50% were reported. No problem happened for those with clopidogrel discontinuation. In Assigned Weights for Diseases, most of our patients had CCI 1–2; some had CCI 3–4 (e.g., controlled DM and COPD). They had no serious disease, leukemia or malignancy. CCI was calculated according to the scoring system established by Charlson et al.21.There were no differences regarding laboratory data at admission time in the 2 groups. Serum amylase levels in both groups (813.24 ± 109.73 U/L and 825.37 ± 96.56 U/L) were more than 9 times upper limit of normal (40-85U/L)), and serum lipase (753.14 ± 139.78 U/L vs. 765.31 ± 23.21 U/L, P = 0.5160) was also more than 4 fold upper limit of normal (0–160U/L). There was no differences regarding Ranson's score (0.84 ± 0.99vs. 0.86 ± 1.02, P = 0.754).

Therefore, patients based on laboratory data and Ranson's score, had diagnosed with mild to moderate gallstone pancreatitis. Also, regarding the Revised Atlanta criteria, they were classified into mild to moderate categories.There was no significant difference between the two groups regarding the need for ERCP after cholecystectomy (43 vs. 40, P = 0.67) (Table 2).

**Table 2:** Mean ± SD of laboratory data at admission time in the two groups.

Variable	Very early (G1)	Late (G2)	P value∗
Ranson's score	0.84 ± 0.99	0.86 ± 1.02	0.754
WBC (/μL)	12000 ± 1610	11610 ± 1820	0.101
BUN (mg/dL)	10.23 ± 1.56	9.90 ± 1.47	0.123
Amylase (U/L) serum	813.24 ± 109.73	825.37 ± 96.56	0.399
lipase (U/L)) serum	753.14 ± 139.78	765.3 ± 123.21	0.516
Total bilirubin (mg/dL)	3.16 ± 0.85	3.16 ± 1.02	0.791
Bilirubin (direct) (mg/dL)	2.12 ± 0.88	2.13 ± 1.00	0.942
ALT (U/L)	366.13 ± 104.73	379.44 ± 114.93	0.366
AST (U/L)	372.57 ± 89.30	368.98 ± 99.41	0.727
ALP(U/L)	341.97 ± 156.93	310.43 ± 166.31	0.161
CRP (mg/L)	110.49 ± 22.27	113.28 ± 21. 5	0.355
LDH (U/L)	446.13 ± 127.29	430.80 ± 128.7	0.389
glucose (mg/L)	100.95 ± 19.48	103.48 ± 21.44	0.375

∗ Independent sample T test.

In the 208 patients, the surgical site infection (SSI) rate was 3.3% (n = 7) so considered as Clavien-Dindo classification of 2 for (SSI) and 0.5%as Clavien-Dindo III for (biliary leak). No Clavien grade IV complication occurred during the investigation. Concerning the complexity of the procedures, according to the Austrian Chamber of Physicians (ERCP = grade 4, cholecystectomy grade 6which expect to have mean length of stay of 9 days) there were no differences regarding peri-operative complications and conversion rate to open surgery (5.7% vs6.7%; p = 0.825).

No choledochal injury happened. We had no recurrent biliary events in G1, but there were 4 cases in the pre-operative interval in G2, including gallstone-related symptoms (recurrent abdominal pain, raised serum amylase), 0% vs. 4%, P < 0.0001.

Two patients in each group had relapse of mild ABP during follow up that was treated medically. There was one case of biliary leak that stopped after ERCP (Table 3).

**Table 3:** Complications of surgery in the two groups of cholecystectomy.

Variable	Very Early	Late early	P value
surgical site infections	3 (3%)	4 (4%)	P = 1∗
bile leak	1 (1.1%)	0 (0%)	P = 1∗
Mortality rate	0 (0%)	0.0%)	NS
Conversion	6 (5.7%)	7 (6.7%)	P = 1∗
ERCP and ES	43 (41%)	40 (19%)	0.67∗∗

∗ Fisher's Exact test.

∗∗ Chi-square test.

We had no postoperative respiratory failure, or local or systemic complication, however a few patients had transient renal insufficiency improved immediately after adequate rehydration in both groups.

Majority of stones had passed through CBD during the first episode of pancreatitis (hospital stay and immediately after discharge) so, ERCP was performed in 83patients (40%), G1 = 43cases, (21%) and G2 = 40cases (19%). None of patients in the early group and 15 patients (14%) in the delayed group underwent pre-cholecystectomy ERCP and esphicterotomy.

## 6 Discussion

Optimal timing of cholecystectomy has not precisely defined, and there is still negative conception in some gastroenterologists about early operation [3, 8, 20, 21, 22]. Surgeons have believed that cholecystectomy during index admission exposed them with a difficult dissection due to inflammatory tissue and edema caused by pancreatitis, and possible increasing surgical complications and conversion rate. However, recent studies confirmed that early cholecystectomy does not increase intraoperative complications [6, 23, 24, 25].

Shir Li Jee [10], in a study on early cholecystectomy within 6 days showed no increase in overall conversion rate, duration of surgery, and morbidity rate. In our study, surgical site infection (SSI) was 3.3% with no difference between the two groups and was lower than others studies (3.3% vs. 7.29%) [27] and on the basis of the Clavien-Dindo classification, it is compatible with Grade IIIa, with a morbidity rate of 13.8% [19]. Patients with higher Clavien scores have severe complications and longer length of hospital stay [19]. Seventy-three percent of our patients were in ASA-I and CCI scores of 1–2 and the rest were in ASA II, with CCI scores of 3–4. Lower rate of SSI in our patients may be related to lower ASA scores and CCI score. In a study conducted by Masoorkhan, SSI in ASA-I was 3.58% and in ASA II-III was16.95% [28]. So, it may be concluded that ABP does not influence the SSI rate.

The results of the present study showed no significant difference in conversion rate between the two groups, 6 patients (5.7%) vs. 7 (6.7%) patients. Also, peri-operative complication rates in the two groups were the same, which showed no significant difference. The causes of conversion in our study included unclear anatomy, hemorrhage in the surgical field, intra-abdominal adhesion in calot's triangle and perihepatic area.

The results of our study were consistent with other studies that compared cholecystectomy within 48 h with those performed after improvement of abdominal pain and liver enzymes level [27].

Retrospective studies have revealed that the risk of recurrence of biliary events following an episode of ABP, after discharge from hospital and before interval cholecystectomy, is considerable and has been reported between 9% and 60% [26, 30].

We have not recurrent biliary events in early group but in the late group, it was 4%. However, it is more favourable than other studies [10, 11, 29].

Length of hospital stay in the delayed group was longer in our study in comparison to very early group (G1) or those reported in the literature [10, 24, 27, 28, 29], and this may increase the cost of treatment without any advantage.

We have used sonography and MRCP instead of intra-operative cholangiography (IOC) and during follow up, only 2% of the cases in each group had retained stones, which clinically presented with post-cholecystectomy pancreatitis. This relapse may be due to migration of biliary sludge or incomplete sphincterotomy. All of these patients responded to medical treatment. This low relapse rate indicates that ultrasonography plus MRCP may be sufficient for CBD stone detection. It seems that the sonographyplus MRCP can replace IOC with the same efficacy, however it needs further investigations.

In Shir Li study, delayed cholecystectomy had longer overall length of stay because 24% of the cases were re-admitted for recurrent biliary events. This may not be so, if cholecystectomy had been performed early [10].

Many of our patients had abdominal pain and vomiting, as well as elevated serum pancreatic enzymes, however, cholecystectomy was performed before resolution of these findings. So, increased serum pancreatic enzymes or abdominal pain and vomiting may not have adverse effect on final outcome.

Our current policy was to perform ERCP and endoscopic sphincterotomy (ES) in CBD stone immediately after discharge, and preoperative ERCP was limited to patients with a total bilirubin <4 mg/dl without cholangitis and CBD dilatation, which strongly suspects to compacted stones or apparent cholestasis. This was done in the same admission period.

There is still controversy about the role and timing of ERCP in ABP and is another cause of delaying cholecystectomy. On the base of guidelines of American Society for Gastrointestinal Endoscopy, there is no role for early ERCP in management of mild acute pancreatitis, if there is no obviously evidence of a retained stone3. Chang et al. [30] showed that in patients with mild to moderate ABP and retained CBD stones, preoperative or post-operative complication rate of ERCP is the same, however the hospital length of stay and costs were significantly fewer in the postoperative ERCP group.

Conclusion: According to the results of the present study, cholecystectomy within 48 h of admission is safe, time saving and more cost beneficial. There is no difficulty in the technical aspect of the procedure, as well. Conversion rate, duration of surgery, and peri-operative complications are the same. We recommend very early cholecystectomy as standard of care in mild to moderate ABP, although it may need more supporting data and studies.

## Declarations

### Author contribution statement

A. Davoodabadi: conceived and designed the experiments; performed the experiments; analyzed and interpreted the data; contributed reagents, materials, analysis tools or data; wrote the paper.

E. Beigmohammadi: conceived and designed the experiments; performed the experiments; contributed reagents, materials, analysis tools or data; wrote the paper.

H. Gilasi: analyzed and interpreted the data; wrote the paper.

A. Arj, H. Taherinassaj: conceived and designed the experiments; contributed reagents, materials, analysis tools or data; wrote the paper.

### Funding statement

This research did not receive any specific grant from funding agencies in the public, commercial, or not-for-profit sectors.

### Competing interest statement

The authors declare no conflict of interest.

### Additional information

The clinical trial described in this paper was registered at WHO IRCT under the registration number IRCT20180205038627N2.
